# A clinical case report on transcranial low-intensity focused ultrasound neuromodulation for central post-stroke pain

**DOI:** 10.3389/fnins.2025.1686623

**Published:** 2025-11-06

**Authors:** Sijin He, Kaixuan Luo, Xiang Li, Jiajia Duan, Lei Ding, Moxian Chen, Xuan Xu, Xianghua Sun, Lijuan Ao, Xiangjun Feng

**Affiliations:** 1 Department of Rehabilitation, Kunming Municipal Hospital of Traditional Chinese Medicine, Kunming, China; 2 The School of Rehabilitation, Kunming Medical University, Kunming, China; 3 Department of Radiology, Kunming Third People’s Hospital, Kunming, China; 4 Department of Cadre Rehabilitation and Surgery, The First Affiliated Hospital of Kunning Medical University, Kunming, China; 5 Shanghai Yangzhi Rehabilitation Hospital (Shanghai Sunshine Rehabilitation Center), Tongji University School of Medicine, Shanghai, China; 6 Department of Rehabilitation, The First Affiliated Hospital of Kunming Medical University, Kunming, China

**Keywords:** transcranial low-intensity focused ultrasound, central post-stroke pain, neuromodulation, cingulum bundle, stroke

## Abstract

Central post-stroke pain (CPSP) manifests as persistent or intermittent pain following cerebral infarction or hemorrhage and is described as “one of the most agonizing, disabling, and refractory pain syndromes.” Its treatment represents a significant clinical challenge. In this case, we used transcranial low-intensity focused ultrasound (tLIFU), an emerging non-invasive neuromodulation approach distinct from pharmacological and traditional neuromodulation methods, to treat CPSP patients, achieving satisfactory outcomes. This approach may inspire new perspectives on innovative pain management. A 66-year-old male veteran suffered long-term CPSP, with unsatisfactory pain relief from previous paregoric interventions, including transcranial magnetic stimulation (TMS). Blood oxygenation level-dependent functional magnetic resonance imaging (BOLD-fMRI) revealed abnormal activity in a region of interest (ROI) that responded to analgesic medication adjustments. This ROI was anatomically consistent with the cingulum bundle. Given this finding, we used tLIFU to demonstrate deep stimulation of the ROI. Remarkable pain reduction was observed after 1 week of tLIFU neuromodulation, allowing for a slight tapering of the gabapentin dose. The analgesic effects of tLIFU were sustained throughout a 5-month follow-up period, with no adverse events reported. Since day 120, the patient has remained off analgesic medications, and at the 150-day follow-up, BOLD-fMRI revealed a normalized activity pattern in the region of interest (ROI). Additionally, significant clinical improvement was noted in the patient’s emotional state. This case report highlights the potential of tLIFU technology to expand therapeutic options in clinical pain management. Exploratory research into the clinical efficacy and the underlying mechanisms of tLIFU in pain treatment may contribute to a deeper understanding of pain pathogenesis and support the development of novel therapeutic strategies.

## Introduction

Central post-stroke pain (CPSP) manifests as persistent or intermittent pain following cerebral infarction or hemorrhage, described as “one of the most agonizing, disabling, and refractory pain syndromes” (Klit et al., 2009; Henry et al., 2008). The current pathogenesis of CPSP remains unclear, and predominant theories include central sensitization, disinhibition of the ventral posterior lateral thalamic nucleus, dysfunctions in pain signaling pathways, functional alterations in the thalamus and other brain regions, and neurotransmitter imbalances. There is a lack of satisfactory efficacy in existing treatment methods for dealing with CPSP. The non-invasive neuromodulation techniques have gained increasing clinical attention for CPSP treatment in recent years (Cheng et al., 2023; Mohanan et al., 2023). Approaches such as transcranial magnetic stimulation (TMS) and transcranial direct current stimulation (tDCS) aim to alleviate pain by applying pulsed magnetic or electric fields to the cerebral cortex, triggering a cascade of physiological responses. However, both tDCS and TMS suffer from low spatial resolution, with a broad radius of action on the order of centimeters (Kubanek, 2018). Therefore, the primary objective of this study is to explore a non-invasive, precise, and effective neuromodulation method that reduces dependence on pain medications. The emergence of transcranial low-intensity focused ultrasound (tLIFU) technology, which has been demonstrated to exert reversible excitatory or inhibitory effects on neural activity (Darrow, 2019), offers a novel, non-invasive deep neuromodulation approach. By utilizing mechanical energy to achieve non-destructive and reversible neuromodulation of neuronal activity, this technique provides millimeter-level spatial resolution and adjustable focal depth, making it suitable for targeting deep brain structures with high spatial precision (Strohman et al., 2024).

In this case study, a patient with long-term CPSP with unsatisfactory pain relief from prior paregoric interventions, including TMS, underwent tLIFU intervention. Once-daily tLIFU treatment over 7 days led to a significant reduction in pain and mood improvement. Within approximately 5-month post-tLIFU treatment, the patient’s analgesic dosage was substantially reduced and eventually discontinued. After tLIFU treatment, blood oxygenation level-dependent functional magnetic resonance imaging (BOLD-fMRI) indicated the disappearance of abnormal signals in the region of interest (ROI). This case report presents a novel approach to pain management and underscores the potential of tLIFU technology for expanding therapeutic options in clinical practice.

### Case presentation

We present the case of a 66-year-old man who developed generalized cutaneous pain at rest 6 months after cerebral infarction, predominantly on the hemiplegic side (the left side). The pain was described as a burning sensation and was exacerbated by contact with clothing or manual skin palpation, with a Visual Analog Scale (VAS) score of 4. After excluding other causes of pain, such as nociceptive or peripheral neuropathic pain, he was diagnosed with CPSP. Gabapentin 0.3 g three times daily was prescribed for pain relief. By June 2024 (7 months after the stroke), the pain on the non-paralyzed side had resolved, but the hemiplegic side continued to exhibit a burning sensation, aggravated by wearing clothes or touching the skin, with the VAS score remaining at 4. The patient had previously underwent TMS for pain relief but did not achieve effective control. To address the persistent pain, the patient and his family signed an informed consent form in December 2024, voluntarily enrolling in a clinical study approved by the Medical Ethics Committee of Kunming Medical University (Approval No. KMMU2024MEC133) investigating tLIFU for pain treatment.

**Figure 1:**
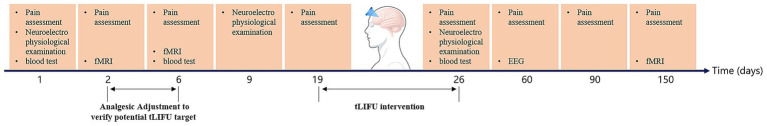
Treatment protocol for the study.

**Figure 2:**
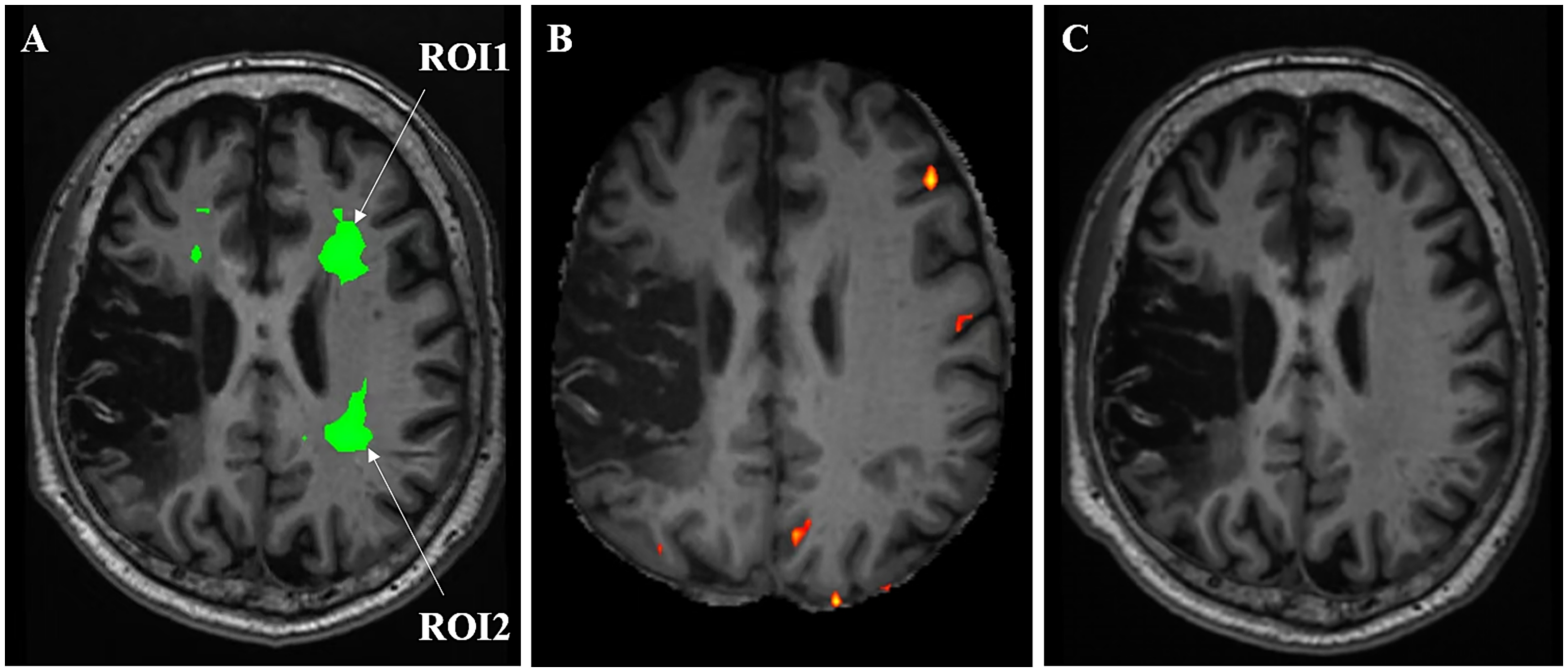
Changes in BOLD-fMRI of ROIs before **(A)** and after **(B)** analgesic drug adjustment and at day 150 follow-up **(C)**.

**Figure 3:**
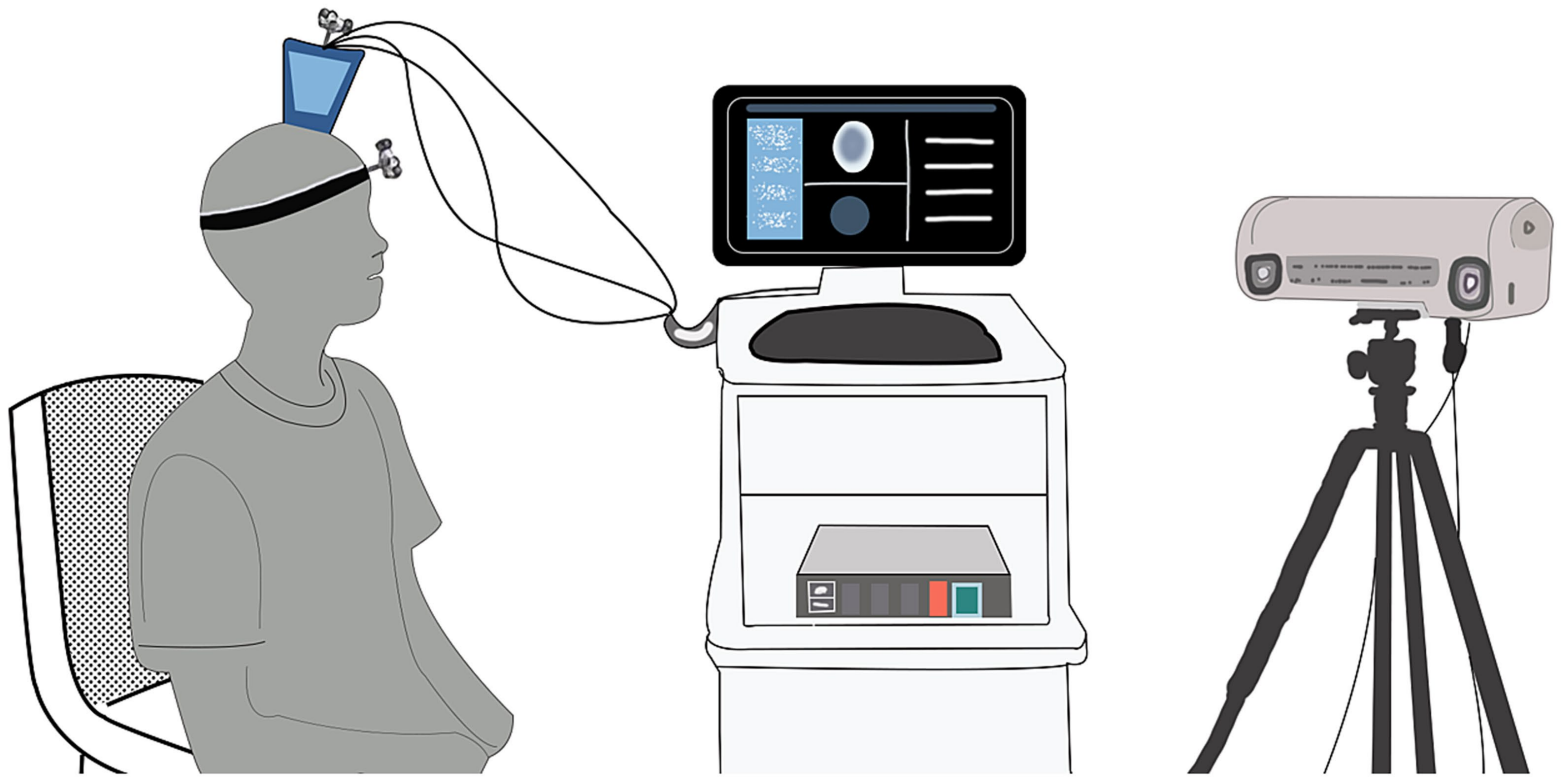
Schematic diagram of tLIFU therapy.

**Figure 4:**
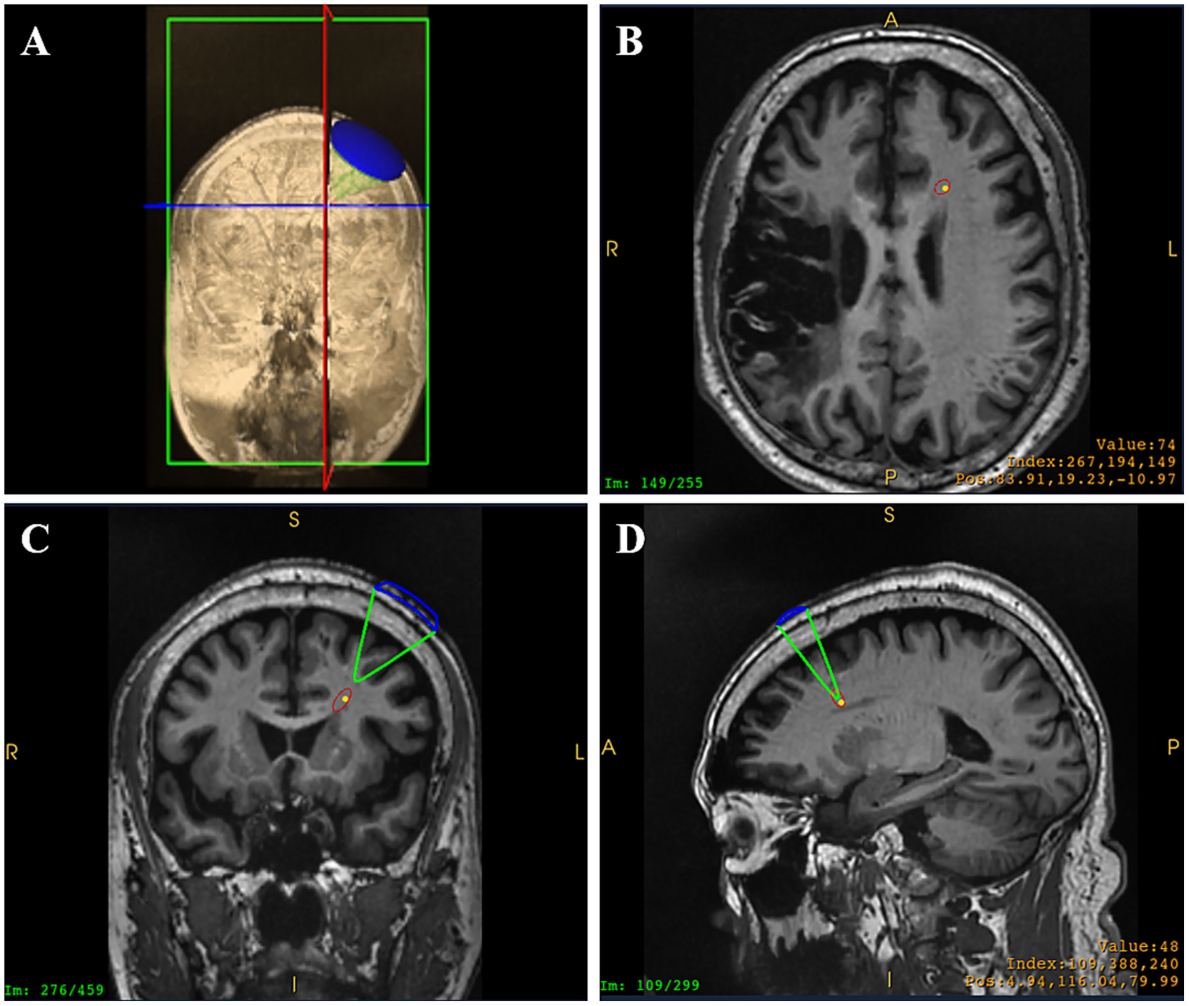
Transducer placement **(A)** and real-time monitoring of tLIFU focus in the horizontal plane **(B)**, the coronal plane **(C)**, and the sagittal plane **(D)** within the therapeutic session (the yellow dot represents the preset neuromodulation target, and the red ellipse represents the focus of the ultrasound transducer. A, anterior; F, feet; H, head; I, inferior; L, left; P, posterior; R, right).

On 31 December (day 1) 2024, blood biochemistry, neuroelectrophysiological, and pain scale assessments were performed (treatment protocol, see Figure 1). On 1 January 2025, when the patient’s CPSP reached a VAS score of 6, BOLD-fMRI revealed two areas of abnormal signal in the patient’s left cerebral hemisphere: region of interest 1 (ROI1) and region of interest 2 (ROI2). From January 2 to 5, ibuprofen sustained-release tablets 0.3 g twice daily were added as an analgesic adjustment for pain relief. Subsequent cranial BOLD-fMRI showed resolution of the abnormal signals in both ROI1 and ROI2 (see Figure 2). In view of (1) the changes in BOLD-fMRI resulting from painkiller adjustment, (2) our prior animal study demonstrating that tLIFU modulation of the anterior cingulate cortex (ACC) effectively alleviated chronic neuropathic pain in mice (Feng et al., 2021), and (3) the proximity of ROI1 to the ACC on the healthy side (the left ACC), ROI1 was therefore selected as the target for tLIFU treatment in this patient. From January 5 to 18, 2025, gabapentin 0.3 g three times daily was prescribed for pain management without using other analgesics. During this period, the patient’s VAS scores fluctuated between 4 and 6, with daily pain attack frequency of 7–8 episodes, each lasting 30 min to 1 h. To preclude interference effects, no other neuromodulation techniques besides tLIFU have been administered to this patient since November 2024. The first neuromodulation via the tLIFU system (GreenValley BrainTech Medical Technology Corporation) with a single-element focused ultrasonic transducer (F0050A03) was carried out on January 18, 2025 (schematic diagram, see Figure 3). The surface of the transducer is circular, with a focal length of 46.8 mm and a focal spot size of 4.80 mm * 4.70 mm * 38.29 mm. The depth of the focal point can be adjusted by modifying the collimator attached to the transducer. To minimize the impact of the thickness of the human skull, the range of tLIFU fundamental frequency that is often used is 250–650 kHz (Cox et al., 2025). A study from our team indicated that tLIFU stimulation with acoustic pressure below 2 MPa is safe for C57 mice (Wang et al., 2022) and that tLIFU stimulation with a duty cycle (DC) of 1.6%, a spatial-peak time-average intensity (ISPTA) of 113.47 mw/cm^2^, and a spatial-peak pulse-average intensity (ISPPA) of 28.37 w/cm^2^ improved social interaction and stereotyped behavior in a boy with autism spectrum disorder (Cheng et al., 2025). Therefore, tLIFU parameters in this study were as follows: the fundamental frequency was 0.5 MHz, the peak-to-peak acoustic pressure was 0.83 MPa, the ISPTA was 53.73 mW/cm^2^, the ISPPA was 5.37 mW/cm^2^, the pulse repetition frequency (PRF) was 200 Hz, and the DC was 1%. The therapeutic session lasted 20 min and was administered once daily for 7 consecutive days. Within the duration of tLIFU therapy, the transducer was placed properly relative to the skull, and its focus of the transducer was aligned with the target site and monitored in real-time to achieve precise neuromodulation (Figure 4). The final tLIFU intervention was completed on 24 January 2025, by which time the dose of gabapentin was tapered to 0.3 g twice daily, with no concomitant use of other analgesics or topical analgesic patches. Clinical assessments demonstrated significant reductions in pain, anxiety, and depression symptoms (see Table 1). Electromyography (EMG) revealed normalized motor conduction in the left tibial nerve and restored sensory conduction in the left ulnar nerve. The immediate electroencephalography (EEG) at the end of the last tLIFU treatment detected epileptiform discharges during photic stimulation (see Table 2). No treatment-emergent adverse events were observed during the therapeutic course, such as blood biochemistry abnormalities, neurological deficits, dizziness, headache, or epileptic seizures. Follow-up at 2-month post-tLIFU neuromodulation demonstrated sustained control of pain and emotional symptoms, with no significant exacerbation of limb pain during rehabilitation exercises. Gabapentin maintenance dose remained at 0.3 g twice daily, without supplementary analgesic or topical analgesic patches. No subjectively reported adverse events were documented, and the EEG showed an absence of epileptiform discharges. At 3-month follow-up, the analgesic efficacy persisted with the continued absence of patient-reported adverse events (see Tables 1, 2). At the 150-day follow-up, the patient had discontinued analgesics for 1 month, with no significant worsening observed in pain or mood (see Table 1), and BOLD-fMRI revealed no abnormal signal in ROIs (Figure 2).

**Table 1:** Pain and psychological assessment results of the patient.

	Baseline (1st day)	Post treatment with gabapentin and ibuprofen (6th day)	Pre-tLIFU intervention (19th day)	Post-tLIFU intervention (25th day)	Follow up (60th day)	Follow up (90th day)	Follow up (150th day)
VAS	6	2	6	2	2	2	3
LANSS	22	22	22	8	8	6	8
Pain detect questionnaire	12	9	11	7	6	4	6
SF-MPQ	21	15	19	10	9	6	8
GPS	64	59	62	31	29	21	23
HAMA	14	9	14	4	4	2	3
HAMD	15	10	14	7	6	4	4
Number of daily pain episodes	7–8	1–2	7–8	1–2	1–2	0–1	0–1
Duration of each pain episode	30 min^−1^ h	10–15 min	30 min^−1^ h	4–10 min	4–10 min	4–6 min	4–10 min
Analgetic interventions	Gabapentin 0.3 g tid; ibuprofen and codeine phosphate tablets; rTMS; loxoprofen sodium gel patch	Gabapentin 0.3 g tid; ibuprofen sustained-release tablets 0.3 g bid	Gabapentin 0.3 g tid; loxoprofen sodium gel patch	Gabapentin 0.3 g bid	Gabapentin 0.3 g bid	Gabapentin 0.3 g bid	Discontinue analgesics for 30 days

GPS, Global Pain Scale; HAMA, Hamilton Anxiety Scale; HAMD, Hamilton Depression Rating Scale; LANSS, Leeds Assessment of Neuropathic Symptoms and Signs; SF-MPQ, Short-Form McGill Pain Questionnaire; VAS, Visual Analog Scale.

**Table 2:** Neuroelectrophysiological assessment.

	Baseline (1st day)	Post-treatment with gabapentin and ibuprofen sustained-release tablets combination therapy (9th day)	Post-tLIFU intervention (24th–25th day)	Follow up (60th day)
EEG	*Dominant rhythm*: all leads predominantly display medium-low amplitude 6–8 Hz alpha-theta mixed activity with fair amplitude modulation and rhythmic organization.	*Dominant rhythm*: all leads predominantly exhibit medium-low amplitude 6–8 Hz alpha-theta mixed activity with adequate amplitude modulation and rhythmic regulation.	*Dominant rhythm*: all leads demonstrate abundant 6, 8–10 Hz alpha-theta waves with poor rhythmic regulation.	*Dominant rhythm*: all leads predominantly display medium-low amplitude 6–8 Hz alpha-theta mixed activity with fair amplitude modulation and rhythmic organization.
	*Slow waves*: all leads show intermixed abundant medium-low amplitude 6–7 Hz theta waves.	*Slow waves*: all leads demonstrate abundant intermixed medium-low amplitude 6–7 Hz theta waves.	*Slow waves*: all leads exhibit frequent medium-amplitude 6 Hz theta waves.	*Slow waves*: all leads show abundant intermixed medium-low amplitude 6–7 Hz theta waves.
	*Fast waves*: all leads contain sporadic low-amplitude 16–18–20 Hz beta waves.	*Fast waves*: scattered low-amplitude 16–18–20 Hz beta waves observed across all leads.	*Fast waves*: occasional low-amplitude 17–20 Hz beta waves sporadically observed across leads.	*Fast waves*: all leads contain sporadic low-amplitude 16–18–20 Hz beta waves.
	*Pathological waves*: no epileptiform discharges observed.	*Pathological waves*: no epileptiform discharges identified.		*Pathological waves*: no epileptiform discharges observed.
	*Photic response*: alpha rhythm demonstrates significant suppression upon eye opening with restoration upon eye closure.	*Photic response*: alpha rhythm shows significant suppression (>70% amplitude reduction) during eye opening, with restoration upon eye closure.	*Photic response*: epileptiform discharges triggered during photic stimulation.	*Photic response*: alpha rhythm demonstrates significant suppression (>50% amplitude reduction) upon eye opening, with restoration upon eye closure.
	*Hyperventilation provocation*: no abnormal activity elicited.	*Hyperventilation provocation*: noncompliance with procedure (unable to perform adequate hyperventilation).	*Hyperventilation provocation*: noncompliance (patient unable to cooperate with procedure).	*Hyperventilation provocation*: no abnormal activity elicited.
	*Photic stimulation*: no driving response or paroxysmal activity noted.	*Photic stimulation*: no photoparoxysmal response or driving rhythm abnormalities detected.	*Photic stimulation*: no photoparoxysmal response or driving abnormalities noted.	*Photic stimulation*: no driving response or paroxysmal activity noted.
	*Brain electrical activity mapping*: abnormal theta-band power distribution across cerebral regions.	*Brain electrical activity mapping*: abnormal theta-band (4–7 Hz) power distribution noted in all cerebral regions.	*Brain electrical activity mapping*: abnormal theta-band (4-7 Hz) power distribution observed in all cerebral regions.	*Brain electrical activity mapping*: abnormal theta-band (4–7 Hz) power distribution across cerebral regions.
	*Amplitude characteristics*: all leads demonstrate high-medium-low amplitude complexes with bilateral symmetry and adequate amplitude modulation.	*Amplitude characteristics*: all leads display high-medium-low amplitude complexes with preserved bilateral symmetry and satisfactory amplitude modulation.	*Amplitude characteristics*: medium amplitude complexes present in all leads with suboptimal amplitude modulation.	*Amplitude characteristics*: all leads demonstrate high-medium-low amplitude complexes with bilateral symmetry and adequate amplitude modulation.
EMG	1. Mild injury to the left peroneal nerve (involvement of motor fibers, demyelinating injury), mild injury to the left tibial nerve (involvement of motor and sensory fibers, demyelinating injury of motor fibers, loss of sensory fiber axons), mild injury to the left median nerve and left ulnar nerve (involvement of sensory fibers, loss of axons). 2. Injury to the proximal nerves or nerve roots of the bilateral median nerves, bilateral ulnar nerves, and bilateral tibial nerves. 3. Impaired autonomic nerve function in the limbs.	1. Injury to the left peroneal nerve and left tibial nerve (involvement of motor fibers, demyelinating injury), mild injury to both ulnar nerves (involvement of sensory fibers, loss of the axons of the left ulnar nerve, demyelinating injury of the right ulnar nerve). 2. Injury to the proximal nerve of the right median nerve, the left tibial nerve or the nerve heel. 3. Impaired autonomic nerve function in the limbs.	1. The left peroneal nerve was injured (motor fiber involvement, demyelinating injury), the right ulnar nerve was slightly injured (sensory fiber involvement, axonal loss), and the left ulnar nerve was normal. 2. Impaired autonomic nerve function in the four limbs.	–
Brainstem auditory evoked potential	Bilateral brainstem auditory pathways demonstrate normal functional integrity.	Bilateral brainstem auditory pathways demonstrate normal functional integrity.	Bilateral brainstem auditory pathways demonstrate normal functional integrity.	–
Visual evoked potential	Bilateral visual pathway dysfunction	Bilateral visual pathway dysfunction	Bilateral visual pathway dysfunction	–

## Discussion

One of the key aspects in the treatment of CPSP using non-invasive neuromodulation technology is the selection of modulation targets. A meta-analysis (Lizi et al., 2024) has demonstrated that neuromodulation of the M1 region can reduce pain intensity, while it has limited efficacy in alleviating patients’ anxiety, depression, or improving quality of life. In this case, the CPSP patient experienced exacerbated pain during movement, with pain sensations triggered upon the anticipation of movement initiation, which subsequently led to the development of anxiety and depressive symptoms. It is clear that M1 is not the optimal target for tLIFU intervention. In this case study, pain assessments were conducted before and after adjusting oral analgesic medications. A comparative analysis of brain BOLD-fMRI data acquired before and after analgesic adjustment revealed positive signal changes in two regions (ROI1 and ROI2). The region closer to the ACC of the healthy side, namely ROI1, was selected as the tLIFU target. A review of the literature and consultations with colleagues in radiology and neurology confirmed that the anatomical locations of both ROI1 and ROI2 correspond well to the cingulum bundle (Bubb et al., 2018; Kollenburg et al., 2025). As a key white matter pathway, the cingulum bundle not only interconnects the frontal, parietal, and medial temporal lobes but also links these regions to the subcortical nuclei, serving as a critical hub for integration. The functions of the cingulum bundle are involved in executive control, emotion, pain, and episodic memory (Bubb et al., 2018). Research indicates that structural abnormalities in the cingulum bundle of adolescents with chronic headache are closely associated with post-traumatic stress symptoms and reduced amygdala volume, suggesting that damage to the structure of the cingulum bundle may promote pain chronification (Miller et al., 2021). Furthermore, abnormalities in the white matter of the cingulum bundle are also associated with impaired cognitive function, particularly deficits in attention and executive function (Hermens et al., 2022). In this case, the primary reasons for selecting the cingulum bundle on the healthy side as the target were not only because the abnormal signal area identified by BOLD-fMRI was located there but also because of our considerations regarding the compromised structural integrity of the functional brain areas on the affected hemisphere and the safety issue of tLIFU neuromodulation on that side. Therefore, the cingulum bundle on the healthy side was deemed an appropriate choice. A possible explanation for why modulating the healthy side (left) cingulum bundle significantly alleviated the pain on the patient’s paralyzed side is inter-hemispheric inhibition. According to the current understanding, the role of inter-hemispheric inhibition is to support contrast-enhancing and integrative functions by co-opting the capacities of the two cerebral hemispheres than to permit the suppression of one hemisphere by another (Carson, 2020). Therefore, it is possible to influence the function of the ipsilateral body by regulating the functional area in one hemisphere. Such examples can be found in neuromodulations. Verin and Leroi (2009) demonstrated that repetitive TMS on the hemisphere of the healthy side of patients with poststroke dysphagia, which resulted in the improvement of swallowing coordination. Another study from Park et al. (2013) also highlights the benefits of 5 Hz high-frequency repetitive TMS on the contra-lesional pharyngeal motor cortex for post-stroke dysphagic patients. Notably, after the application of tLIFU treatment to the left cingulum bundle, a significant alleviation of ipsilateral pain and improvement in mood were observed in the CPSP patient, and the positive outcomes were well-maintained at the 5-month follow-up, by which time the patient had already stopped taking painkillers for 1 month. These findings suggest that tLIFU targeting the cingulum bundle for CPSP treatment·may represent a novel therapeutic attempt in pain management.

The biophysical effects of ultrasound can broadly be divided into thermal and non-thermal effects (Krishna et al., 2018). High-intensity focused ultrasound (HIFU) leverages its thermal effects to achieve tissue ablation for therapeutic purposes and has been clinically applied to treating conditions such as essential tremor, Parkinson’s disease, and brain tumors (Elias et al., 2013; Ikeda et al., 2016; Kaplitt et al., 2024; Krishna et al., 2023; MacDonell et al., 2018). The non-thermal effects of ultrasound, including mechanical pressure, radiation force, and cavitation, offer numerous possibilities for neuromodulation. Low-intensity focused ultrasound (LIFU) can modulate neuronal activity by influencing mechanosensitive voltage-gated ion channels or neurotransmitter receptors and even by altering membrane conformation (Zhu et al., 2023; Kubanek et al., 2016; di Biase et al., 2019; Krasovitski et al., 2011). Moreover, by modulating central nervous system (CNS) plasticity (Wang et al., 2022; Baek et al., 2018; Mesik et al., 2024), LIFU could thereby exert a profound influence on brain function. In the study by Baek et al. (2018), the lateral cerebellar nucleus (LCN) of the stroke mouse model underwent tLIFU with an excitatory sonication parameter, and motor-evoked potentials (MEPs) were generated in both forelimbs. LCN stimulation via tLIFU significantly enhanced sensorimotor recovery and decreased the level of brain edema as well as tissue swelling in the affected hemisphere. In another study conducted by Mesik et al. (2024), tLIFU stimulation of the visual thalamus produced long-term depression (LTP) of thalamocortical synapses in the visual cortex of adult mice. Moreover, reversible blood–brain barrier opening and drug delivery achieved by tLIFU are under investigation for the treatment of neurodegenerative diseases and brain tumors (Luo et al., 2022; Wang et al., 2022; Brighi et al., 2022). Although numerous studies have investigated the mechanisms and therapeutic effects of tLIFU, the mechanisms underlying tLIFU remain to be fully elucidated.

The analgesic effect of tLIFU has been proven. In the preliminary study, our team investigated the analgesic effect of tLIFU stimulation targeting the ACC in a chronic constriction injury (CCI) mouse model. The results demonstrated that tLIFU significantly increased both short-term and long-term mechanical pain thresholds and reduced pain sensitivity (Feng et al., 2021). Clinical evidence supports the analgesic efficacy of tLIFU. In a study by Shin et al. (2023), 11 patients with refractory neuropathic pain underwent tLIFU neuromodulation targeting the ACC. The frequency used in the abovementioned study was 250 kHz, ISPTA was 0.72 W/cm^2^, duty cycle varied from 50 to 70%. The results demonstrated significant reductions in both VAS and Short-Form McGill Pain Questionnaire (SF-MPQ) scores. The therapeutic effects were maintained at the 3-month follow-up, with no serious adverse events reported during the study period. The patient cohort included various etiologies: spinal cord injury (four cases), compressive myelopathy (three cases), post-spinal surgery syndrome (three cases), and cauda equina syndrome (one case). The study, however, did not include any case of CPSP. This study presents a novel clinical case utilizing tLIFU to modulate the cingulum bundle for CPSP management. Notably, we implemented optimized treatment parameters with increased stimulation frequency and reduced session duration. Encouragingly, the results demonstrated significant clinical improvements: reduction in pain assessment scale scores with a smaller dose of painkiller, decreased depression/anxiety scale ratings, markedly fewer pain episodes with shortened duration, and substantial mood enhancement. These therapeutic benefits remained sustained at the time of the 3-month follow-up evaluation. The patient discontinued analgesic at the 120-day mark, and by the 150-day follow-up, no significant worsening in pain or mood was observed. No abnormal signal within the patient’s cingulum bundle region was detected via BOLD-fMRI, providing evidence for the long-term efficacy of tLIFU neuromodulation. The mechanisms by which tLIFU regulates pain may involve modifications to pain-processing brain circuits, pain-related signaling pathways, and neuroplasticity. Kim et al. (2024) demonstrated that tLIFU stimulation at pain-processing brain circuits (e.g., primary somatosensory cortex and insula) significantly altered pain-associated behaviors in mouse models. Analyses of brain electrical rhythms through electroencephalography demonstrated a significant change in noxious heat hypersensitive- and chronic hyperalgesia-associated neural signals following focused ultrasound treatment. Due to the extensive connections of the cingulum bundle with multiple pain circuits, tLIFU that targets the cingulum bundle may modulate its functional activity. This modulation could, in turn, indirectly regulate pain-related network sites—such as the ACC, the lateral dorsal nucleus of the thalamus, and the prefrontal cortex—thereby producing an analgesic effect (Bubb et al., 2018; Kollenburg et al., 2025). Evidence from studies suggests that LIFU could relieve pain by regulating pain-associated signaling pathways. LIFU stimulation of the L4–L5 section of the spinal cord alleviated neuropathic pain by improving the potassium chloride cotransporter 2 (KCC_2_) expression and inhibiting the Calcium/Calmodulin-dependent Protein Kinase IV (CaMKIV)–KCC_2_ pathway in rats with peripheral nerve injury. (Liao et al., 2021). Moreover, LIFU stimulation of the dorsal root ganglion with LIFU ameliorated pain responses through the GABA-CGRP pathway (Lin et al., 2025). tLIFU, additionally, may modulate pain by influencing neuroplasticity. tLIFU that targeted ACC significantly reversed aberrant central plasticity caused by CCI surgery and improved the pain response in mice (Wang et al., 2022). However, due to the complex pathogenesis of pain, the analgesic mechanisms of LIFU remain to be further investigated.

Interestingly, we serendipitously observed that the patient’s motor conduction of the left tibial nerve, as well as the sensory conduction of the left ulnar nerve, were normalized following tLIFU treatment, as EMG displayed. The cingulum bundle has a complex structure composed of multiple fiber tracts, including thalamocortical projections, cingulate gyrus projections, and projections to the prefrontal as well as posterior parietal cortices, which coordinate networks among these brain regions. Data from MRI and PET studies indicate that its functions encompass a wide range of processes, including sensory processing, memory, spatial function, reward, cognition, emotion, visceral motor activity, and endocrine regulation (Kollenburg et al., 2025). In this case, the EMG improvements may be attributed to the natural recovery processes on the one hand, and on the other hand, we hypothesize that tLIFU-mediated cingulum bundle modulation could alleviate local central sensitization and pain-related abnormal discharges, thereby improving the sensory function of the left ulnar nerve. Furthermore, pain relief may regulate cognitive-emotional signals that influence motor decision-making, resulting reduced abnormal muscle activity then potentially facilitates motor conduction recovery of the left tibial nerve. Specifically, tLIFU-mediated neuromodulation of the CNS may not only exert targeted effects on specific brain regions but could also influence peripheral nerve function via potential central-peripheral communication mechanisms. This provisional hypothesis, however, requires rigorous validation through further investigations.

Notably, EEG performed immediately after the final tLIFU treatment captured epileptiform discharges during photic stimulation (see Table 2). However, follow-up EEG at 2 months showed no epileptiform activity, and the patient had no history of epileptic seizures since the stroke onset. We considered several aspects for the following reasons: first, focused ultrasound is a mechanical wave, and the acoustic radiation force may activate mechanosensitive ion channels. The imbalance between excitation and inhibition in the CNS after cerebral infarction is primarily associated with ion channels. Alterations in ion channels may contribute to abnormal synchronous discharges in local neurons (Kubanek et al., 2016; Steinlein, 2014), thereby inducing epileptiform discharges, which could represent an immediate effect of tLIFU. The modulation of neuronal activity by focused ultrasound, however, is reversible, which may also explain the absence of epileptiform discharges observed at the second follow-up. Second, the occurrence of stroke may increase the risk of seizures. Studies have shown that cerebral infarction in the middle cerebral artery supply area carries a high risk of post-stroke epilepsy (Zou et al., 2015). Third, gabapentin may exert dual effects of pain relief as well as seizure prevention in this case. The dosage reduction of gabapentin following tLIFU treatment might potentially elevate the risk of epileptiform discharges observed on EEG. However, it is concerning whether the epileptiform discharges observed via EEG are associated with tLIFU application. A previous study has reported that deep-brain stimulation (DBS) implanted in the ACC for refractory pain management induced epileptic seizures or even status epilepticus in some participants, regardless of whether the DBS was turned on or off. This finding was attributed to excessive stimulation intensity. Reducing the DBS intensity to a safe threshold that did not induce seizures resulted in diminished analgesic efficacy (Nüssel et al., 2022; Maslen et al., 2018). In this case, the ISPPA of tLIFU targeting the cingulum bundle was 5.37 mW/cm^2^, far below the U.S. Food and Drug Administration (FDA) recommendation of ISPPA ≤ 190 W/cm^2^. Moreover, the ISPTA was 53.73 mW/cm^2^, significantly lower than the FDA recommendation of ISPTA ≤ 720 mW/cm^2^ (US Food and Drug Administration, 2019). In a study demonstrated by Bubrick et al. (2024), six patients with refractory epilepsy underwent tLIFU treatment, among whom one patient experienced a typical epileptic seizure during the first session. When the duty cycle was reduced from 50 to 18.3%, no further seizures occurred, and no adverse events were observed during follow-up. In the current study, the duty cycle was 1%, indicating an extremely low likelihood of triggering epileptic seizures. At the 150-day follow-up, some of the patients’ indices showed slight rebound. We consider two possible reasons for this: first, many of these indices are influenced by the subjectivity of both the patient and the assessor; second, at the time of assessment, the patient had already discontinued analgesic medication for 1 month. Therefore, such fluctuations appear acceptable, and longer-term tracking is necessary to determine the definitive effects of tLIFU. There are several crucial points to acknowledge here. One is that this study employs an optical navigation system. While it offers greater operational ease than MRI-guided navigation, the system displays the tLIFU focal point in real time as a “theoretical” position, calculated based on the patient’s T1-weighted MRI images. The actual ultrasound propagation path is influenced by factors such as the skin, underlying tissues, and the skull. Future technological advances, including the development of ultrasound phased arrays, hold promise for optimizing the clinical application of tLIFU (Hynynen and Jones, 2016). Another crucial point to acknowledge is that, while the modulatory effect of tLIFU on pain has been reported (Feng et al., 2021; Lizi et al., 2024; Shin et al., 2023; Liao et al., 2021; Lin et al., 2025) and is supported by the compelling therapeutic effect observed in our CPSP case, its efficacy for complex regional pain syndrome or other drug-resistant pain types remains unclear. Furthermore, given the subjective nature of pain and its multitude of influencing factors, randomized controlled trials are warranted to elucidate the efficacy and mechanisms of tLIFU for pain management.

## Conclusion

As an emerging non-invasive neuromodulation technique, tLIFU has demonstrated significant potential for pain management. In this case, the patient suffered from long-term CPSP, a chronic central neuropathic pain condition that impacts both physiological and psychological wellbeing. Prior pharmacological analgesia and TMS intervention failed to achieve satisfactory outcomes. Consequently, we attempted tLIFU neuromodulation for the purpose of better pain management. At the beginning, we acquired the patient’s cranial BOLD-fMRI when the pain intensity peaked and abnormal signals were observed in ROIs, which were anatomically consistent with the cingulum bundle. Following the analgesic medication adjustment, we repeated the BOLD-fMRI when the pain subsided to its lowest level and the signal abnormality in ROIs disappeared. The tLIFU therapy under the guidance of optical navigation, subsequently directed at ROI1, led to preliminary clinical improvement. This individualized tLIFU therapy achieved preliminary success: the patient reported significant pain reduction at 1-week post-treatment with a reduced dose of painkiller, and the analgesic effects were sustained as revealed by the follow-ups. At the 120-day mark, the patient discontinued analgesics, and at the 150-day follow-up, pain remained controlled, and the region of ROIs exhibited a normalized activity pattern, as revealed by BOLD-fMRI. Additionally, the patient exhibited clinically significant improvement in mood. To our knowledge, this study represents the first case report of tLIFU-based precise therapy for chronic CPSP. However, prospective studies validating the efficacy and safety of varying tLIFU parameters in chronic neuropathic pain patients remain lacking. Future studies are warranted to expand the clinical applications of tLIFU and to benefit a broader patient population.

## Data Availability

The original contributions presented in the study are included in the article/supplementary material, further inquiries can be directed to the corresponding authors.

## Glossary

ACC: anterior cingulate cortex
BOLD-fMRI: blood oxygenation level dependent functional magnetic resonance imaging
CaMKIV: Calcium/Calmodulin-dependent Protein Kinase IV
CCI: chronic constriction injury
CNS: central nervous system
CPSP: central post-stroke pain
DBS: deep brain stimulation
EEG: electroencephalography
EMG: electromyography
FDA: Food and Drug Administration
GPS: Global Pain Scale
HAMA: Hamilton Anxiety Scale
HAMD: Hamilton Depression Rating Scale
HIFU: high-intensity focused ultrasound
ISPPA: spatial-peak pulse-average intensity
ISPTA: spatial-peak time-average intensity
KCC_2_: potassium chloride cotransporter 2
LANSS: Leeds Assessment of Neuropathic Symptoms and Signs
LCN: lateral cerebellar nucleus
LIFU: low-intensity focused ultrasound
LTP: long-term potentiation
MRI: magnetic resonance imaging
PRF: pulse repetition frequency
ROI: region of interest
SF-MPQ: Short-Form McGill Pain Questionnaire
tDCS: transcranial direct current stimulation
tLIFU: transcranial low-intensity focused ultrasound
TMS: Transcranial magnetic stimulation
VAS: Visual Analog Scale
